# Crassulacean acid metabolism as a continuous trait: variability in the contribution of Crassulacean acid metabolism (CAM) in populations of *Portulacaria afra*

**DOI:** 10.1016/j.heliyon.2017.e00293

**Published:** 2017-04-13

**Authors:** Lonnie J. Guralnick, Kate Gladsky

**Affiliations:** Department of Biology, Roger Williams University, Bristol, RI 02809 USA

**Keywords:** Plant biology, Evolution, Ecology

## Abstract

*Portulacaria afra* L. is a dominant facultative CAM species growing in the Southeastern Cape of South Africa. *P. afra* is well adapted to regions of the Spekboom thicket in areas of limited and sporadic rainfall. *P. afra* populations occur in isolated drainages. We hypothesized the utilization of CAM would vary in the different populations in response to rainfall and temperature gradients. Carbon isotope composition can be used to determine the contribution of CAM in leaf tissue. *P. afra* leaves of populations were analyzed in transects running south to north and east to west in locations from the coast to elevations of 1400 m. Carbon isotope values ranged from −16.1‰ in Plutosvale to −21.0‰ to −22.7‰ in Port Alfred and Grahamstown populations respectively with some values reaching −25.2‰. These values indicated an estimated variable contribution of the CAM pathway ranging from 23% to almost 60%. The results indicate a much greater range of variability than previously reported. The carbon isotope values showed no direct correlation with rainfall or maximum or minimum day/night temperatures in the summer or winter for the different locations. The results indicated the microclimate may play a more significant role in determining CAM utilization. We present evidence that CAM is a continuous trait in *P. afra* and CAM is operating continuously at low levels during C_3_ photosynthesis which may explain the high variability in its carbon isotope composition. *P. afra* populations illustrate a large phenotypic plasticity and further studies may indicate genotypic differences between populations. This may be valuable in ascertaining the genetic contribution to its water use efficiency and possible use in engineering higher water use efficiency in C_3_ plants. The results revealed here may explain *P. afra*’*s* ability to sequester carbon at high rates compared to more mesic species.

## 1 Introduction

Crassulacean acid metabolism (CAM) is a metabolic and anatomical adaptation that is characterized by net nocturnal carbon dioxide uptake with a temporal separation of the C_4_ and C_3_ pathway (Osmond, 1978; Ting, 1985). The CO_2_ is fixed by Phosphoenolpyruvate carboxylase (PEPCase), converted to malate and stored as malic acid in the vacuole during the night. In the subsequent light period, the malate is decarboxylated to release CO_2_ for utilization by Rubisco in the C_3_ cycle. CAM plants typically have a leaf mesophyll anatomy with primarily spongy parenchyma cells with a large central vacuole (Gibson, 1982). The uptake of CO_2_ at night with the stomata closed during the day results in an increased water use efficiency when compared to C_3_ and C_4_ plants (Black, 1973). CAM has evolved in at least 35 different plant families including six aquatic families and over 343 genera (Borland et al., 2011).

The δ ^13^C values can be used as an investigative tool to determine if plants are utilizing C_3_, C_4_, or CAM photosynthesis. The δ ^13^C values are indicative of whether CO_2_ is fixed by Rubisco or are indicative of the utilization PEPCase as the main carboxylating enzyme during growth. C_3_ plants have carbon isotope composition values closer to −27‰ while C_4_ plants have values which are more positive at −11‰ (Black, 1973). CAM plants can have values ranging from −14‰ to −25‰ depending on the overall contribution of nocturnal CO_2_ uptake (Winter and Holtum, 2002). Obligate CAM species will have values much closer to the values of C_4_ plants due to primarily fixing CO_2_ at night with a small contribution of exogenous daytime CO_2_ uptake in the early morning and late afternoon. Facultative CAM species vary along the C_3_ to C_4_ continuum depending on the overall utilization and contributions of CAM to the carbon balance of the plants. Thus, plants which predominantly use CAM will have values closer to C_4_ species while plants which have predominantly daytime CO_2_ uptake will have an isotope signal in the range of C_3_ species.

*Portulacaria afra* L., a succulent member of the Didereaceae (formerly of the Portulacaceae), is native to South Africa and commonly found in semi-arid areas (Cowling and Proche Vlok, 2005). *Portulacaria afra*, called elephant’s food locally in South Africa has small succulent leaves. The plant can grow from 2–5 m in height as a large woody shrub or small tree (Oakes, 1973; Baran, 1999). The branches show dichotomous branching and the leaves are opposite one another. The leaves of the plants last at least one growing season or more (Guralnick et al., 1984b; Baran, 1999). *P. afra* currently occupies approximately 1.7 million hectares (Mills et al., 2005) in the eastern and southeastern Cape. The rainfall can occur throughout the year but spring and fall have heavier rainfall maxima with some summer rainfall in the Eastern Cape. Precipitation can range from 250 to 750 mm per year. *P. afra* is the dominant vegetation in parts of the Spekboom veld and in some areas can compose up to 90% of the stand. In these areas *P. afra* can form a closed canopy shrubland (Mills et al., 2005). *P. afra* grows primarily in regions that are frost free but it is found in regions where the temperature has reached −6 °C (Oakes, 1973). This ability to withstand frost is aided by the density of the *P. afra* stands (Palmer and Pitman, 1961).

*Portulacaria afra* has been shown to be a facultative Crassulacean acid metabolism species (Guralnick and Jackson, 2001). A facultative CAM species can perform daytime CO_2_ uptake, traditional C_3_ photosynthesis, but utilize the CAM pathway during times of water stress. *Portulacaria afra* was first shown to have nocturnal CO_2_ uptake and a large acid fluctuation when water stressed (Ting and Hanscom, 1977; Hanscom and Ting, 1978). Later studies of *P. afra* have indicated the CAM response is seasonal and related to long day photoperiods, showing more CAM activity during the summer months when temperatures are higher (Guralnick et al., 1984a, b). However, *P. afra* is also able to utilize the C_3_ pathway with predominantly daytime photosynthesis in the cooler months and thus shows a faster growth rate than obligate CAM plants (i.e., Jade plant, *Crassula argenta*) which can only take in exogenous CO_2_ during the night period and in the early morning or late afternoon of the daytime period.

*P. afra* is a facultative CAM species and utilizes CAM under a variety of environmental conditions. It has been shown that *Portulacaria afra* primarily utilizes the CAM pathway during its growth in South Africa as noted by its reported measurements of the δ^13^C composition (^13^C/^12^C ratios) of −17.1‰ (Mooney et al., 1977). The δ^13^C composition measured by Mooney et al. (1977) was taken from one population in the Cape and this measurement was taken over 35 years ago. Mills et al. (2005) found a δ^13^C range of −17.4 to −20.5‰ for plants in the Fish River region in the Eastern Cape at an elevation of 300–500 m.

*Portulacaria afra* has a widespread distribution and many of the populations are isolated in different drainages from other *P. afra* populations. The phenotypic variation in growth habitat may be an indicator of genotypic variation in the different populations. We hypothesized the contribution of CAM to the overall growth could vary among different populations depending on the particular environmental conditions to which the populations are acclimated. Those populations which have the least amount of rainfall would be expected to have a greater overall contribution of CAM to the δ^13^C composition. Our goal was to measure the δ^13^C composition of different *P. afra* populations in the Eastern Cape. These results will help to determine which populations have greater water use efficiencies and could be used for restoration. We report our findings for the δ^13^C composition of ten populations of *P. afra*.

## 2 Materials and methods

### 2.1 Climate and plant material

**Fig. 1:**
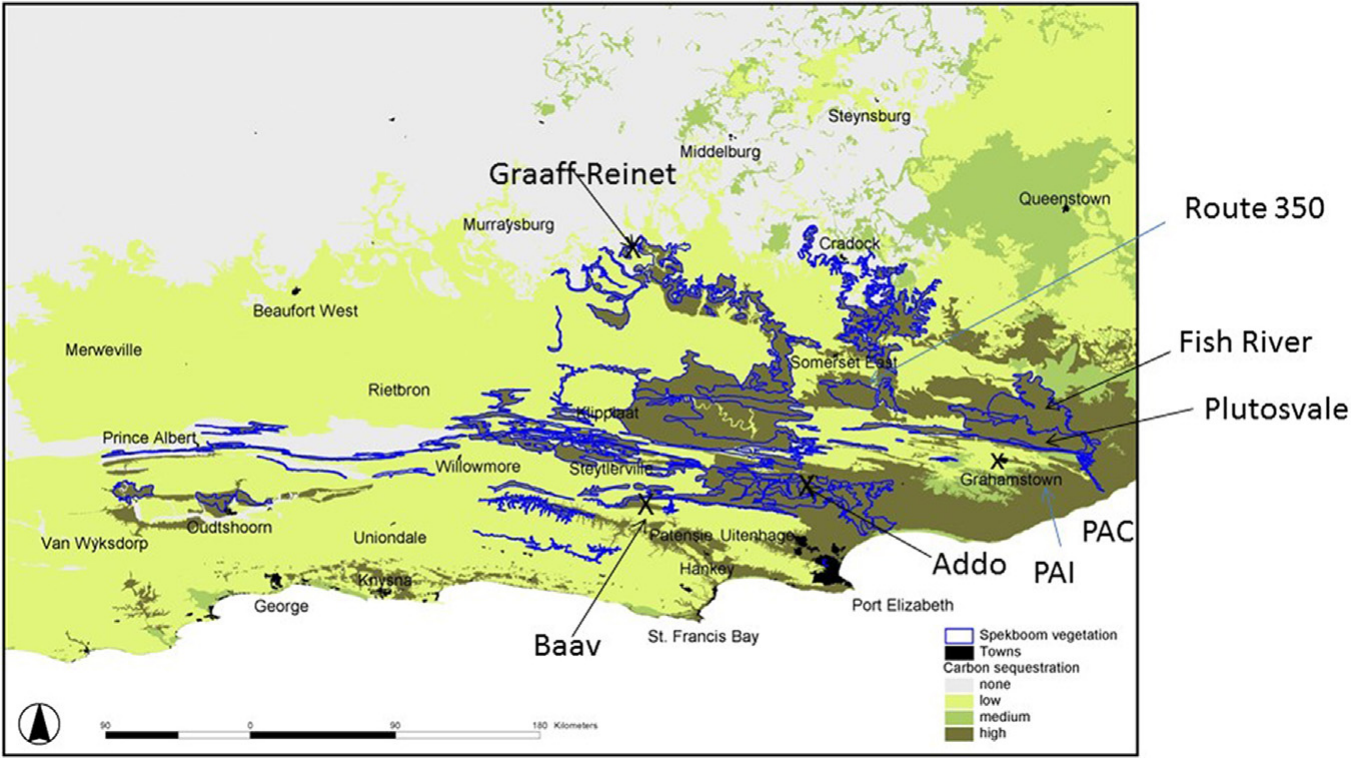
Map of location of collecting sites of various populations of *Portulacaria afra*. The Blue represents the distribution of the Spekboom Thicket. Baav (Baviaanskloof); Addo Elephant National Park; Grahamstown; Route 350; Fish River; Plutosvale; Port Alfred; Graaff −Reinett. Map Courtesy of Mike Powell.

Temperature and rainfall characteristics of various locations near the sample sites in the Eastern Cape are shown in Table 1. Average daily maximum summer temperatures are similar for the region as a whole while winter temperatures show greater variation within the Eastern Cape. Populations were sampled in east to west transect and a south to north transect (Fig. 1). Rainfall varies from 214 mm in Baviaanskloof (westernmost sample; 24°61′W) to 837 mm in the Coastal region of Port Alfred (southernmost sample; 33°41′S). The elevation varied from sea level in Port Alfred to 500–600 m in Plutosvale and Grahamstown to 1400 m in Graff-Reinet (northernmost sample; 32°14′N). The easternmost sample near the Fish River Reserve was taken at 26°42′E. The summer maximum temperature in the different areas ranges from 32 °C in Addo Elephant National Park, 30 °C in Graaff-Reinet to 27 °C in Port Alfred. The winter maximum temperature varies from 16 °C in Baviaanskloof to 23 °C in Port Alfred and 25 °C in Graaff-Reinet. Baviaanskloof has the coolest average winter temperature at 3 °C. Leaf samples of different populations of *Portulacaria afra* (L.) Jacq. were collected in the summer during the day (March 10-March 15, 2013 and January 3-January 18, 2014) and frozen at −80 °C until processed and assayed. Mature leaf samples (second and third leaves from the apical position) were randomly collected from different plants at approximately noon from sun and shade position. New leaf growth was collected from apical positions on the branches. Reproductive tissue was also collected if the plants in the population were flowering.

### 2.2 Carbon isotope analysis

The δ ^13^C was determined from frozen leaf and flower specimens, dried in an oven at 65 °C and sent to Washington State University (College of Sciences Stable Isotope Core; http://www.isotopes.wsu.edu) for analysis (Guralnick et al., 2008). Sample sized ranged from 5–36 for populations. Estimates of Nighttime CO_2_ uptake were calculated using equations from Winter and Holtum (2002). Data were analyzed using a one-way ANOVA; a Tukey post-hoc comparison was utilized to measure any significant differences (p < 0.05) between populations.

## 3 Results

### 3.1 Carbon isotope composition

The δ ^13^C varied among the populations with average values ranging from −17.7 to −22.7‰ (Table 2). The most positive values were found in Plutosvale and Route 350 which were intermediate distances along both the south/north and east/west transect. The Plutosvale population was significantly less negative than the Addo, Baviaanskloof, and the Port Alfred coastal populations. The most negative isotope values were found in Grahamstown and the Graaff-Reinet populations. The populations near Addo Elephant National park, Baviaanskloof, the Port Alfred inland population, and the population north of Grahamstown were intermediate between the Grahamstown and Plutosvale populations showing δ^13^C values ranging from −19.0 to −19.6‰ (Table 2). The population near Addo Elephant National park was −19.8‰ which was intermediate between the Port Alfred coastal and Plutosvale. The high Altitude population of Graaff-Reinet (1400 m) was similar in values to the Port Alfred Coastal population. The average δ ^13^C value of all populations was −19.6‰. An analysis of the carbon isotope values showed no correlation with summer or winter maximum temperatures, summer day/night differentials, or average rainfall.

The carbon isotope composition values for new growth were not significantly different from that of the mature leaves for all but one population (Table 3). In the Fish River population, new growth was significantly more negative than the mature leaves of the population. Only the Addo population had flowers and the samples were significantly less negative than the mature leaves of the population (Table 3).

Carbon isotope samples were analyzed from species growing together in the same area for comparison. *P. afra* showed an isotope value of −19.6‰ (Table 4). The C_3_ species of *Plumabago auricalata* had a δ^13^C of −28.7‰ and *Pappea capensis* showed a δ^13^C of −26.4‰. The succulent species of *Crassula cultrata* showed a δ^13^C of −17.1‰ (Table 4). Given that the C_3_ species showed a range of δ^13^C values of −26.0 to −30.2‰, we then estimated nocturnal contribution of overall CO_2_ uptake. The populations varied in their nighttime contribution from ∼22.75% in the Grahamstown population to over ∼50% in the Plutosvale and Route 350 population (Table 5, Winter and Holtum, 2002). The other populations ranged from 30.2 to 47.4% for an estimated overall contribution nocturnal CO_2_ uptake. Individual plant samples showed a much higher contribution of nocturnal CO_2_ uptake, in some cases up to 60% of the overall carbon balance. On the other end of the spectrum, the contribution of nighttime carbon uptake was reduced to ∼10% of the overall balance.

## 4 Discussion

The expected results for plants in the Eastern Cape would be that contribution of CAM would vary in accordance with either rainfall abundance, increased day/night temperature differentials or day/night maxima temperatures. The results found in this study did not support this hypothesis. The mature leaf samples from populations collected in the various locales of the Eastern Cape showed variation from −17.7‰ to −23.1‰ with individual values ranging from −16.4‰ to −25.2‰ for *P. afra*. Our results indicate a larger range of values than reported by Mills et al. (2005) for *P. afra*. Mooney et al. (1977) previously measured *P. afra* and reported a value of −17.5‰ from plants measured at Plutosvale (near Grahamstown). The results reported here from Plutosvale illustrated that the ∂^13^C values are still in a similar range after 37 years. This is under conditions of increasing atmospheric CO_2_ concentrations from 330 ppm to ∼400 ppm. The results reported for populations of *P. afra* are similar to the range of values detected in three species of *Clusia* from Panama (Holtum et al., 2004). New leaf tissue showed similar values to the mature leaf tissue except for one population. This result is an indicator that most carbon for the growing leaves is imported from the mature leaf tissue as reported by Winter and Holtum (2002). The reproductive tissue was more CAM like and may have been produced during a period of reduced soil water.

There appears to be a linear relationship between the carbon isotope values and the proportion of daytime and nighttime CO_2_ fixation (Winter and Holtum, 2002; Winter et al., 2015). Our results confirmed the C_3_ growing with *P. afra* fell in the C_3_ range enabling estimation using ∂^13^C of nighttime contribution of CO_2_ uptake, We estimated the nighttime contribution of CO_2_ uptake of *P. afra* populations using equations from Winter and Holtum (2002). We hypothesized the nighttime contribution of CO_2_ uptake would follow trends in the rainfall patterns. The Port Alfred population had a more C_3_ like value with an estimated contribution of ∼30% nighttime CO_2_ uptake (Table 5). The Addo population which still has somewhat of a coastal influence, but with less rainfall than Port Alfred, showed more of intermediate C_3_ and CAM value with an estimated 42% contribution of nighttime CO_2_ uptake. The more CAM like values were found in Route 350 and Plutosvale populations which grow further inland and show approximately a 50% contribution on nocturnal CO_2_ uptake. These regions have higher summer maxima day and night temperatures which are more conducive to CAM photosynthesis. Their values fell in the range in which 50–60% of the carbon uptake and indicates nighttime CO_2_ fixation by PEPCase (Winter and Holtum, 2002). Based on the range of δ ^13^C values, it appears the contribution of the CAM pathway can vary from 20% to 60% with an average for some populations between 50 and 60 percent. This is large range for the contribution of nocturnal CO_2_ uptake and indicates a large variation of CAM photosynthesis within the *P. afra* species.

The range of δ ^13^C values measured in this study did not show a significant correlation between the rainfall and temperature conditions that the various populations were growing in. There were slight trends, such as the Port Alfred coastal population which grows under milder conditions during both winter and summer and receives the most precipitation of all of the populations (Table 1). The results indicate the populations may have distinct microclimates which caused the contribution of CAM to vary widely even though populations are not too distant from one another. The Grahamstown population with a δ ^13^C of −22.7‰ is 20–25 km from the Plutosvale population (which is east of Grahamstown) which had a δ ^13^C of −17.7‰ and receives more rainfall. A population 20 km north of Grahamstown (Hellspoort Valley) had a δ ^13^C of −19.6‰ and was similar to the Port Alfred inland population which was south of Grahamstown. The Baviaanskloof population which is the furthest west had a δ ^13^C of −19.1‰ and has less rainfall than Plutosvale. This region may have a slight influence of the Western Cape climate as it is close to the transition zone of the Mediterranean climate and winter rainfall which is more conducive to C_3_ photosynthesis. Those populations further east have more of a summer rainfall pattern and experience a winter drought. Plants with summer rainfall still experience high evapotranspiration and midday loss of turgor pressure resulting in the utilization of CAM (Guralnick and Ting, 1988). This may explain the more CAM like signal in plants in summer rainfall areas.

Most CAM and C_3_-CAM species fall into bimodal distribution of carbon isotope values ranging from −27‰ to −14‰ Winter and Holtum (2002). The results of Winter and Holtum (2002) showed there are relatively few species found in the range from −19‰ to −22‰. The range of δ ^13^C values observed in the different populations of *P. afra* are interesting in that they fall in within the range where the majority of most facultative CAM species are conspicuously absent. The overall δ ^13^C average of the ten populations was −19.6‰ and falls clearly in the range where few species are found. These are values are estimated to have a contribution of nighttime CO_2_ uptake in the range of 40–50% of the overall carbon balance. It is believed this range is not phenotypically adaptable to most species and which is why there is a bimodal distribution of CAM species (Winter et al., 2015). Most species Winter and Holtum (2002) observed were either predominantly C_3_ or showed more than a 50% contribution of CAM to the carbon balance of the species.

**Fig. 2:**
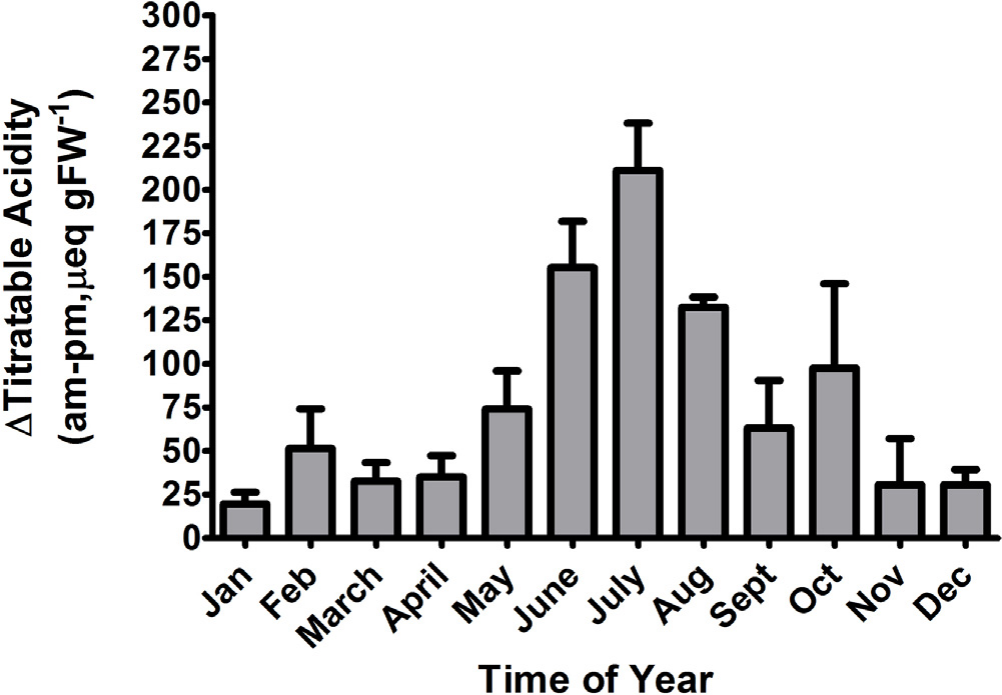
The monthly average change of titratable acidity (AM- PM) of well-watered *Portulacaria afra* plants grown in Southern California in the Northern Hemisphere. Summer months are June, July, and August. (n = 4-9, error bars indicate 1 SEM). Data is taken from Guralnick (1983) and Guralnick (1987).

*P. afra* is well adapted to maximize its carbon uptake with its ability to shift between C_3_ and CAM on a daily basis. In *P. afra* CAM is operating continuously ranging from C_3_ to CAM-Cycling (refixation of respiratory CO_2_) to CAM and CAM-idling depending on environmental conditions (Guralnick et al., 1984b). This may account for its carbon isotope signal to fall in the range from −19‰ to −22‰. The utilization of CAM varies during the course of the year (Guralnick et al., 1984b). *P. afra* has previously reported values ranging from −27.5‰ (Sternberg et al., 1984), −25.2‰ (Neales, 1975) and −23.4‰ (Guralnick and Ting, 1986) dependent on growth conditions. The value of −23.4‰ reported by (Guralnick and Ting, 1986) were from well-watered plants growing outdoors in a Mediterranean climate of Southern California and CAM was utilized extensively for 3–4 months during the summer (Guralnick and Ting, 1988). In addition, *P. afra* exhibits high levels of housekeeping PEPCase activity enabling the plant to utilize CAM during the winter months and then shows an increased induction of PEPCase activity during the summer months (Guralnick and Ting, 1988). During the winter months, *P. afra* exhibits an average acid fluctuation in the range from 20 to 34 μeq gFW^−1^ which is typical of plants that are recycling respiratory CO_2_ at night (Fig. 2). Thus, *P. afra* exhibits what is termed low-level CAM (Winter et al., 2015) during the season when predominantly C_3_ photosynthesis is observed. During the summer months, *P. afra* exhibits an average acid fluctuation in the range from 125 to 210 μeq gFW^−1^. Thus, *P. afra* exhibits a strong CAM signal with substantial amounts of nocturnal CO_2_ uptake and carbon gain. In every month *P. afra* exhibits some measurable acid fluctuation which indicates within this species, CAM is a continuous trait.

The population results of this study are similar to results of population studies of *Sedum wrightii* reported by Kalisz and Teeri (1986). They found δ^13^C values ranging from −13.8 to −22.9‰. Further research indicated genetic differences of populations where plants from the more arid environments appeared to have a higher degree of tolerance to drought and more significant utilization of CAM than the other populations (Gurevitch et al., 1986). Population level studies of *P. afra* will be critical to the understanding of its role in the Spekboom thicket because it is a dominant species and can account for over 90% of the plant cover. In Graaff-Reinet, *P. afra* occurs at a plant density of 440 plants/Ha (Guralnick, unpublished data) and other areas can even have higher plant densities. In comparison, the woody species of *Clusia* has densities ranging from 40–100 plants/HA in their community but do not dominate the landscape like *P. afra* (Quevedo et al., 2013).

The research presented here illustrates different populations show a marked phenotypic plasticity in their utilization of CAM and growth patterns, which is presumed to be environmentally related. We have started initial genetic screening of the populations and there are indications there may be genetic differences in the populations (Guralnick and Gladsky unpublished data). More information will be needed to determine if these differences may have some photosynthetic components. Plants from different populations have also been transplanted to a common garden for more detailed physiological comparisons. We have initiated microclimatic studies looking at soil variables and water relations in the different populations. This in turn may lead to populations that may be better adapted for restoration due to increased water use efficiency. The water use efficiency (WUE) of C_3_ *P. afra* appears to be higher than a typical C_3_ plant and is in the range of 1.2–8.3 mmol CO_2_/mol H_2_O and may be related to its leaf anatomy and reduced mesophyll airspace of 5% (Borland et al., 2009; Guralnick unpublished data). This may be important as researchers are working to genetically engineer higher water use efficiencies in C_3_ plants that would be more typical of CAM species (Borland et al., 2009). The results here suggest that *P. afra* may be an ideal candidate to study the molecular aspects of facultative CAM metabolism (Winter and Holtum, 2014).

## 5 Conclusions

We provide evidence which indicated a much greater range of variability in the contribution of CAM than has been previously identified in areas where *P. afra* is found. We also extend the altitudinal range to 1400 m in which *P. afra* is found and performing CAM. We are continuing our studies on the contribution of the CAM pathway to the overall carbon balance and water use efficiency in *Portulacaria afra* and its possible role in carbon sequestration in South Africa. The overall evidence supports that CAM is a continuous trait in *P. afra*.

## Declarations

### Author contribution statement

Lonnie J. Guralnick: Conceived and designed the experiments; Performed the experiments; Analyzed and interpreted the data; Contributed reagents, materials, analysis tools or data; Wrote the paper.

Kate Gladsky: Performed the experiments; Analyzed and interpreted the data; Wrote the paper.

### Funding statement

Research was supported in part from a Rhode Island-INBRE fellowship awarded to Kate Gladsky. Support was also provided by an award from NSF-EPSCoR.

### Competing interest statement

The authors declare no conflict of interest.

### Additional information

No additional information is available for this paper.

## Figures and Tables

**Table 1:** Annual rainfall and average maximum and minimum temperatures in regions of the Eastern Cape.

Location	Rainfall (mm)	Summer Avg Daily Max/Min °C (January)	Winter Avg Daily Max/Min °C (July)
Addo Elephant National Park^a^	>445	32/15	18/5
Baviaanskloof^b^	214	29/14	16/3
Graaff-Reinet^c^	395	30/17	25/5
Grahamstown^b^	444	29/14	19/5
Plutosvale^b^	665	29/17	22/7
Port Alfred^b^	837	27/18	23/10

a www.sanspark.org/parks/addo/tourism/climate.php.

b www.worldweatheronline.com.

c www.Graaffreinet.com/climate.

**Table 2:** Carbon Isotope values of populations of *Portulacaria afra* in the Eastern Cape.

Location	δ^13^C Isotope Value Average^x^ (‰)	Range
Addo Elephant National Park (n = 13)	-19.0(0.3)^a^	-16.5/20.0
Baviaanskloof (n = 16)	-19.1(0.1)^ab^	-18.6/-19.9
Fish River (Route 67) (n = 36)	-18.1(0.1)^abc^	-16.2/-19.9
Graaff-Reinet (n = 10)	-21.3(0.6)^deg^	-16.8/-23.0
Grahamstown (n = 31)	-22.7(0.2)^d^	-20.0/-25.2
Grahamstown (n = 6)^y^(Hellspoort Valley)	-19.6(0.8)^abcef^	-17.0/-21.6
Plutosvale (n = 19)	-17.7(0.4)^cf^	-16.0/-22.0
Port Alfred coast (n = 6)	-21.0(0.2)^de^	-20.4/-21.5
Port Alfred Inland (n = 7)^z^	-19.5(0.9)^abceg^	-16.4/-23.1
Route 350 (n = 5)	-17.5(0.2)^abcf^	-16.9/-17.9

x Populations followed by the same letter are not significantly different from each other (p < 0.05 level). Numbers in parentheses are 1 SEM.

y Grahamstown population 25 km north of city center near Hellspoort Valley turnoff.

z Port Aflred Inland population (35 km from Grahamstown).

**Table 3:** Carbon Isotope values of new growth in populations of *Portulacaria afra* in the Eastern Cape.

Location	δ^13^C New Growth (‰)	δ^13^C Mature Leaves^a^ (‰)
Addo Elephant National Park (n = 5) Addo (Reproductive tissue (n = 3)	-17.9(0.4) -16.1(0.1)*	-19.0
Fish River (Route 67) (n = 4)	-20.5(0.3)*	-18.1
Graaff-Reinet (n = 5)	-21.7(0.4)	-21.3
Grahamstown (n = 5)	-23.0(0.7)	-22.7
Plutosvale (n = 4)	-17.4(0.1)	-17.7
Port Alfred coast (n = 4)	-21.0(0.5)	-21.0
Port Alfred Inland (n = 5)^b^	-17.7(0.3)	-19.5

* Samples followed by the asterisk indicate that the tissue is significantly different from mature tissue (p < 0.05 level). Numbers in parentheses are 1 SEM.

b Port Alfred Inland population (35 km from Grahamstown).

**Table 4:** Carbon Isotope Composition of C_3_ species associated with *Portulacaria afra*.

Species	δ^13^C Isotope Value Average^x^(‰)	Range
*Portulacaria afra* (N = 6)	-19.6(0.8)^a^	-17.0–21.6
*Plumabago auricalata* (N = 3)	-28.7(1.0)^b^	-26.9–30.2
*Pappea capensis* (N = 3)	-26.4(0.5)^b^	-25.7–27.4
*Crassula cultrata* (N = 3)	-17.7(0.3)^a^	-17.2–18.2

x Populations followed by the same letter are not significantly different from each other (p < 0.05 level). Numbers in parentheses are 1 SEM.

**Table 5:** Proportion of Nighttime CO_2_ Uptake of *Portulacaria afra* populations in the Eastern Cape.

Location	δ^13^C Isotope Value Average^w^^,^^x^(‰)	Nighttime CO_2_ Uptake (%)
Addo Elephant National Park (n = 13)	-19.0(0.34)^a^	42.6
Baviaanskloof (n = 16)	-19.1(0.09)^ab^	42.1
Fish River (Route 67) (n = 36)	-18.1(0.14)^abc^	47.4
Graaff-Reinet (n = 10)	-21.3(0.57)^deg^	30.2
Grahamstown (n = 31)	-22.7(0.24)^d^	22.7
Grahamstown (n = 6)^y^ (Hellspoort Valley)	-19.6(0.80)^abcef^	39.5
Plutosvale (n = 19)	-17.7(0.39)^cf^	50.1
Port Alfred coast (n = 6)	-21.0(0.18)^de^	31.9
Port Alfred Inland (n = 7)^z^	-19.5(0.93)^abceg^	39.9
Route 350 (n = 5)	-17.5(0.18)^abcf^	50.7

w Estimates of Nighttime CO_2_ uptake calculated using equations from Winter and Holtum (2002).

x Populations followed by the same letter are not significantly different from each other (p < 0.05 level). Numbers in parentheses are 1 SEM.

y Grahamstown population 25 km north of city center near Hellspoort Valley turnoff.

z Port Alfred Inland population (35 km from Grahamstown).

## References

[bib0005] Baran R.J. (1999). *Portulacaria afra* the Elephant’s food or spekboom. arxiv:/www.users.qwest.net/~rjbphx/Portulacaria.html.

[bib0010] Black C.C. (1973). Photosynthetic carbon fixation in relation to net CO_2_ uptake. Ann. Rev. Plant Physiol..

[bib0015] Borland A.M., Griffiths H., Harwell J., Smith J.A.C. (2009). Exploiting the potential of plants with Crassulacean acid metabolism for bioenergy production on marginal lands. J. Exp. Bot..

[bib0020] Borland A.M., Zambrano V.A.B., Ceusters J., Shorrock K. (2011). The photosynthetic plasticity of crassulacean acid metabolism: an evolutionary innovation for sustainable productivity in a changing world. New Phytol..

[bib0025] Cowling R.M., Proche Vlok J.H.J. (2005). On the origin of southern African subtropical thicket vegetation. S. Af. J. Bot..

[bib0030] Gibson A., Ting I.P., Gibbs M. (1982). Anatomy of Succulence. Crassulacean acid metabolism. Proceedings of the Fifth Symposium in Botany.

[bib0035] Guralnick L.J. (1983). Photoperiodic control of the induction of Crassulacean acid metabolism in *Portulacaria afra*.

[bib0040] Guralnick L.J. (1987). The effect of drought on the seasonal shift from C_3_ to CAM photosynthesis in *Portulacaria afra* (L.) *Jacq*.

[bib0045] Guralnick L.J., Jackson M.D. (2001). The occurrence and phylogenetics of Crassulacean acid metabolism activity in the Portulacaceae. Int. J. Plant Sci..

[bib0050] Guralnick L.J., Ting I.P. (1986). Seasonal response to drought and rewatering in *Portulacaria afra* (L.) *Jacq*. Oecologia.

[bib0055] Guralnick L.J., Ting I.P. (1988). Seasonal patterns of water relations and enzyme activity of the facultative CAM plant *Portulacaria afra* (L.) *Jacq*. Plant Cell Environ..

[bib0060] Guralnick L.J., Rorabaugh P.A., Hanscom Z. (1984). Influence of photoperiod and leaf age on Crassulacean acid metabolism in *Portulacaria afra* (L.) *Jacq*. Plant Physiol..

[bib0065] Guralnick L.J., Rorabaugh P.A., Hanscom Z. (1984). Seasonal shifts of photosynthesis in *Portulacaria afra* (L.) *Jacq*. Plant Physiol..

[bib0070] Guralnick L.J., Cline A., Smith M., Sage R. (2008). Evolutionary Physiology: The extent of C_4_ and CAM photosynthesis in the Genera Anacampseros and Grahamia of the Portulacaceae. J. Exp. Bot..

[bib0075] Gurevitch J., Teeri J.A., Wood A.M. (1986). Differentiation among populations of *Sedum wrightii* (Crassulaceae) in response to limited water availability: Water Relations, CO_2_ assimilation, growth and survivorship. Oecologia.

[bib0080] Hanscom Z., Ting I.P. (1978). Response of succulents to plant water stress. Plant Physiol..

[bib0085] Holtum J.A.M., Aranda J., Virgo A., Gehrig H.H., Winter K. (2004). δ^13^C values and Crassulacean acid metabolism in *Clusia* species from Panama. Trees.

[bib0090] Kalisz S., Teeri J.A. (1986). Population-level variation in Photosynthetic metabolism and Growth in *Sedum wrightii*. Ecology.

[bib0095] Mills A.J., Cowling R.M., Fey M.V., Kerley G.I.H., Donaldson J.S., Lechmere-Oertel R.G., Sigwela A.M., Skowno A.L., Rundel P. (2005). Effects of goat pastoralism on ecosystem carbon storage in semiarid thicket, Eastern Cape, South Africa. Austral Ecol..

[bib0100] Mooney H.A., Troughton J.H., Berry J.A. (1977). Carbon isotope ratio measurements of succulent plants in Southern Africa. Oecologia.

[bib0105] Neales T.F., Marcelle R. (1975). The gas exchange patterns of CAM plants. Environmental and biological control of photosynthesis.

[bib0110] Oakes A.J. (1973). *Portulacaria afra Jacq*. −A potential browse plant. Econ. Bot..

[bib0115] Osmond C.B. (1978). Crassulacean acid metabolism: A curiosity in context. Ann. Rev. Plant Physiol..

[bib0120] Quevedo A.A., Schleuning M., Hensen I., Saavedra F., Durka W. (2013). Forest Fragmentation and edge effects of the genetic structure of *Clusia sphaerocarpa* and *C. lechleri* (Clusiaceae) in tropical montane forests. J. Trop. Ecol..

[bib0125] Palmer E., Pitman N. (1961). Trees of South Africa.

[bib0130] Sternberg L.R., Deniro M.J., Ting I.P. (1984). Carbon, Hydrogen, and Oxygen Isotope Ratios of Cellulose from Plants Having Intermediary Photosynthetic Modes. Plant Physiol..

[bib0135] Ting I.P. (1985). Crassulacean acid metabolism. Ann. Rev. Plant Physiol..

[bib0140] Ting I.P., Hanscom Z. (1977). Induction of acid metabolism in *Portulacaria afra*. Plant Physiol..

[bib0145] Winter K., Holtum J.A.M. (2002). How closely do the δ^13^C values of Crassulacean acid metabolism plants reflect the proportion of CO_2_ fixed during the day and night. Plant Physiol..

[bib0150] Winter K., Holtum J.A.M. (2014). Facultative crassulacean acid metabolism (CAM) plants: powerful tools for unraveling the functional elements of CAM photosynthesis. J. Exp. Bot..

[bib0155] Winter K., Holtum J.A.M., Smith J.A.C. (2015). Crassulacean acid metabolism: a continuous or discrete trait?. New Phytol..

